# Hearing loss in aging

**DOI:** 10.1016/S1808-8694(15)31123-X

**Published:** 2015-10-20

**Authors:** Giovana dos Santos Baraldi, Lais Castro de Almeida, Alda Cristina de Carvalho Borges

**Affiliations:** 1 Master's degree on Human Communication Disorders - UNIFESP, and Gerontology Specialist. Speech Therapist; 2 Master's degree on Human Communication Disorders - UNIFESP. Speech Therapist; 3 Doctoral degree on Human Communication Disorders, Adjunct Professor at UNIFESP

**Keywords:** elderly, hearing threshold, presbycusis

## Abstract

**Summary:**

Aging is a natural consequence of a society developing process. The city of São Paulo has almost one million people who are above sixty years of age. Age-related hearing loss equals the total hearing loss resulted from cell degeneration caused by noise exposure, ototoxic agents and the loss caused by disorders and medical treatments.

**Aim:**

To study age-related hearing degeneration by means of higher thresholds and hearing sensitivity measures.

**Materials and Methods:**

Cross-sectional contemporary cohort study in which we assessed 211 elderly patients with mean age of 75.24 years, of whom 61 were females and 150 were males. The subjects were submitted to an interview and a conventional audiometric assessment; and later divided into four groups according to age range.

**Results:**

Significant threshold drop in the four established age groups, decrease in speech recognition ratio, and a significant difference regarding gender.

**Conclusion:**

As age advanced there was a gradual increase in hearing loss, men showed a lower threshold in the 4000Hz frequency when compared to women, and in the speech intelligibility test thre was also a gradual decrease with aging.

## INTRODUCTION

Aging populations are a world trend and are due to an increased life expectancy and reduced mortality and birth rates. The estimated life expectancy at birth in Brazil in 2003 for both sexes reached 71.3 years, a 0.8 year increase compared to the year 2000 (70.5). According to the most recent mortality projection, in 2040 Brazil will have reached a life expectancy at birth of 80 years.^1^ According to the last Census (2000), the elderly population was 5.85% of the population, a 1.02% increase compared to the previous Census in 1991. The aging rate also increased from 13.90% in 1991 to 19.77% in 2000.^2^ The city of Sao Paulo has nearly one million people aged over 60 years, who have contributed and still contribute to the growth of the city.^3^

Aging is a natural consequence of societal development, which in this case appears as increased control over demographic variables: both as more effective family planning (reflected in decreasing fertility rates) and, mostly, increased control over mortality (which results in increased life expectancy). Within this context ongoing studies are essential to deepen our knowledge about the mechanisms responsible for aging and to provide a basis for the actions of planners and executive agents.^4^

According to the World Health Organization (WHO), elderly people are those aged over 65 years. This reference age, however, is valid for developed countries. In developing countries such as Brazil, the age that defines elderly people is 60 years.^5^

The percentage of the population that presents communication difficulties increases gradually with age, compounded by auditory deficiencies and cognitive loss. Hearing loss is the most prevalent sensorial loss in this age group. Brazilian studies have shown that hearing loss affects around 60% of the elderly population residing in Brazil. This variable includes varying degrees of hearing loss and social limitations.^6^

Age-related hearing loss is the sum of hearing losses that result from various forms of physiological degeneration, including losses caused by exposure to noise, ototoxic agents, and losses caused by medical disorders and treatment.^7^ Age-related hearing loss affects around 60% of all people aged over 65 years,^8^ and includes a gradual decline in auditory sensitivity at all frequencies accompanied by a decrease in speech discrimination. There is also a complex decline in central auditory function, evidenced as increased difficulty in abilities such as auditory fusion, auditory attention, auditory judgment, varied behaviors, and reduced auditory closing velocity and auditory synthesis.^9^

Studies have demonstrated that auditory loss has a negative effect on the functional status, on quality of life, on cognitive function, and on the emotional, behavioral and social well-being of elderly persons.^10^

Hearing changes with age include progressive sensorial, neural, and strial hearing loss, decreased cochleal cell support and reduced central neural processing.11 The effects of age on the peripheral and central auditory systems interact with changes such as decreased cognitive support, decreased perception, increased thresholds, decreased speech understanding in noisy and reverberant ambiences, and lowered perception of rapid changes in speech and in sound localization. Studies during the past century have clarified many aspects on the effect of age on the auditory system, but otogerontology faces new challenges in the new millennium. These challenges include developing methods to modulate age-related hearing loss and rehabilitation strategies and interventions to meet all individual hearing-related needs.^11^

Considering the need for studies to deepen our knowledge about the age-related physiological degeneration of the auditory system, the aim of this paper was to investigate these age-related losses using auditory supraliminal and sensitivity tests.

## METHOD

This study was analyzed and approved by the Sao Paulo Federal University Research Ethics Committee according to established guidelines for research on human beings, as defined by the National Health Board regulation CEP number 1266-04.

Our sample included elderly patients referred to us by the UNIFESP Geriatrics and Gerontology Institute, which is located in Sao Paulo. Audiological evaluation was done between January and July 2004. The sample included 211 elderly patients aged between 60 and 99 years; the mean age was 75.24 (SD= 8.96). There were 61 men and 150 women in the sample.

A clinical history was initially collected, including questions about habits considered harmful to hearing, questions on auditory acuity, and hearing function within the social milieu, including: information about difficulty to understand speech in a noisy environment and over the telephone; the need to increase television volume; social deprivation due to hearing loss; the otological history; the presence of tinnitus and dizziness.

Patients then underwent a basic audiological evaluation in an acoustic booth, which included the following procedures:

• Threshold Tonal Audiometry: done using an ANSI69-calibrated Interacoustis Model AC33 audiometer.
• Voice Audiometry: speech recognition threshold (SRT) and the percentage index of speech recognition (PISR).
• Acoustic Immitance Test: Tympanometry and a survey of contralateral acoustic reflexes, done using an Interacoustis Modelo AZ7 Immitanciometer.

Audiometry was classified into grades: mild, moderate, moderately severe, severe, and profound, according to Davis and Silverman (1970).^12^ As suggested in literature, and to improve characterization of the progression of hearing loss with age, individuals were divided into four age groups (60 to 69 years, 70 to 79 years, 80 to 89 years and over 90 years). This criterion was established because the trend is to propose new aging stages based on age and the level of functional independence. Research on aging involving people aged 60 or above, that does not take into account the diversity of control of various resources in people aged 60 and those 20 or 30 years older, may be questioned.^13^

Results were interpreted using ANOVA tests, the test for Equality of Two Proportions, the Chi-squared test, and a descriptive analysis composed by the Confidence Interval. A p-value of 0.05 was used.

## RESULTS

According to the aims of this study, age-related loss of auditory sensitivity may be assessed by obtaining the auditory thresholds for each frequency, and by measuring the percent index of speech recognition. Thus, our results were based on auditory thresholds found for each tested frequency and the speech discrimination values.

## DISCUSSION

The growing number of elderly people and the life expectancy, particularly in southeastern Brazil, means increased comorbidity in this age group. Phonoaudiological studies of auditory disorders in the elderly have increased, and speech therapists are required to keep abreast of the needs of the population. Auditory changes in the elderly range from lowered auditory thresholds to significant difficulties in understanding speech, resulting in communication problems and social isolation. Given the limitations of hearing loss, an early diagnosis becomes essential to reduce the impact on social relationships.

The results of this study show that the frequency of female elderly patients was higher than the frequency of male elderly patients (Table 1); our sample included 150 women and 61 men. This fact may be explained by data from IBGE (the Brazilian Institute of Geography and Statistics) that found more elderly women than men in the Brazilian year 2000 census. The Brazilian elderly population is roughly 13,915,357 people (8.1% of the population), of which 6,309,588 (45.3%) were men and 7,605,769 (54.7%) were women. The average age for both sexes was 75.24 years (Table 2).

**Table 1:** Distribution by gender of individuals evaluated.

	Gender	
	N	%
Male	61	28,9%
Female	150	71,1%
Total	12	100%

**Table 2:** Distribution by age of individuals evaluated.

AGE	
Average	75.24
Mean	75.00
Standard deviation	8.96
Minimum	60
Maximum	99

The audiological profile of our sample showed a prevalence of bilateral sloping high-frequency sensorineural hearing loss (at 4, 6 and 8 kHz) in both ears. These findings were also reported by Roehe et al., Pedalini et al., Gonçalves, and Mota and Mazelova et al.14,15,16,17

An assessment of the degree of hearing loss based on frequency averages at 500, 1000 and 2000 Hz proposed by Davis and Silverman^12^ showed that 32.2% of individuals in our sample had normal hearing, 28% had mild hearing loss, 25.6% had moderate hearing loss, 6.2% had moderately severe hearing loss, 5,7% had severe hearing loss, and 2.4% had profound hearing loss. These findings are similar to those encountered by Pedalini et al., Russo, Katsarkas and Ayukawa, Bacha et al., and Gonçalves and Mota,^15^,^16^, ^17^, ^18^, ^19^, ^20^ who reported preservation of low frequencies in cases of age-related hearing loss. Roehe^14^ et al. commented the expected loss of low frequencies in auditory aging. These findings underline the importance of using a classification system based on various frequencies to establish the degree of hearing loss in the elderly. An example is the classification proposed by Silman and Silverman^21^ that uses an average based on tone thresholds at low and middle frequencies (500, 1000 and 2000 Hz) and high frequency tone thresholds (3000 and 4000 Hz) to establish the degree of hearing loss. Longone and Borges^22^ used the average based on the pure tone threshold at 6000 Hz to classify the degree of hearing loss in the elderly, based on Katz^23^ who reports that individuals with hearing loss at 6000 Hz show communication difficulties in the presence of noise, a frequent complaint in this population group.

**Figure 1:**
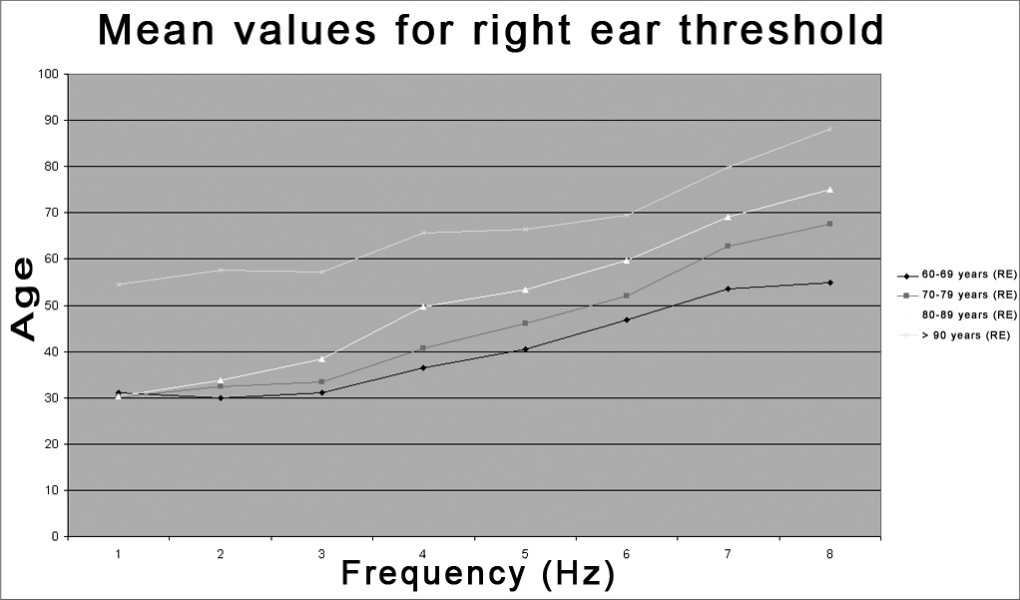
Distribution of threshold averages according to the age group in the right ear (RE) - RE = right ear

We found right ear hearing threshold stability at low frequencies (250, 500, and 1000 Hz) in the first three of four age groups (60-69, 70-79, 80-89, and >90 years), and a significant threshold decline only in the >90 years age group. There was a significant threshold decline at high frequencies (2, 3, 4, 6, and 8 kHz) in all four age groups, with significant differences between groups, where the >90 years age group had the highest hearing losses at all frequencies (Table 3 and Figure 1). Bess et al.^9^ reported similar results, showing that hearing loss in individuals aged over 60 years is seen mostly at high frequencies, particularly those over 1000Hz.

**Table 3:** Distribution of the threshold average according to the age group - Right Ear (RE).

Threshold average RE								
Age group	250Hz	500Hz	1000Hz	2000Hz	3000Hz	4000Hz	6000Hz	8000Hz
60-69 years	31,15	30,00	31,10	36,53	40,51	46,78	53,56	54,83
70-79 years	30,11	32,50	33,37	40,71	46,14	51,96	62,72	67,61
80-89 years	30,38	33,72	38,33	49,62	53,33	59,62	69,10	75,00
> 90 years	54,52	57,62	57,14	65,71	66,43	69,52	79,76	88,10

**Figure 2:**
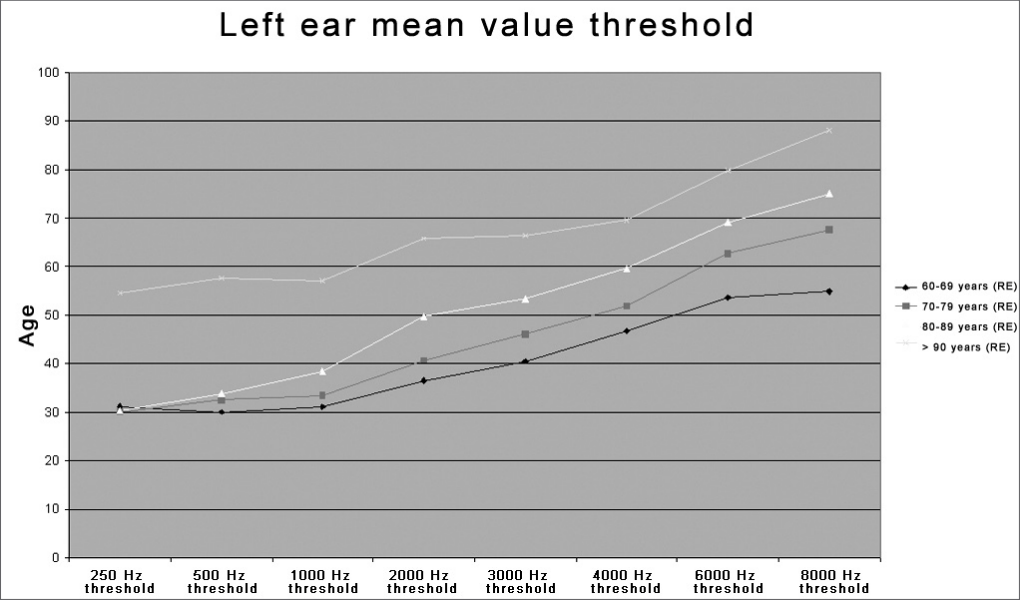
Distribution of threshold averages according to the age group in the left ear (LE) - LE = left ear

Similar values were observed in the left ear for all four age groups (60-69, 70-79, 80-89, and >90 years). Table 4 and Figure 2 show low frequency (250, 500 and 1000 Hz) auditory threshold stability in the first three age groups, and a significant threshold decline only in the >90 years age group. There was a significant high frequency (2, 3, 4, 6, and 8 kHz) threshold decline in all four age groups. These results show that low frequency thresholds do not change significantly until the 80-89 year age group, and is significantly compromised over age 90 years. High frequency thresholds are lowered even at less advanced age. These findings are similar to those reported by Russo18 who investigated 169 elderly men and women with presbyacusis, divided into 5 age groups (5-year intervals), where hearing loss increased gradually with age and varied according to frequency, with greater loss at higher frequencies in both sexes.

**Table 4:** Distribution of the threshold average according to the age group - Left Ear (LE).

Threshold average LE								
Age group	250Hz	500Hz	1000Hz	2000Hz	3000Hz	4000Hz	6000Hz	8000Hz
60-69 years	31,15	30,00	31,10	36,53	40,51	46,78	53,56	54,83
70-79 years	30,11	32,50	33,37	40,71	46,14	51,96	62,72	67,61
80-89 years	30,38	33,72	38,33	49,62	53,33	59,62	69,10	75,00
> 90 years	54,52	57,62	57,14	65,71	66,43	69,52	79,76	88,10

**Figure 3:**
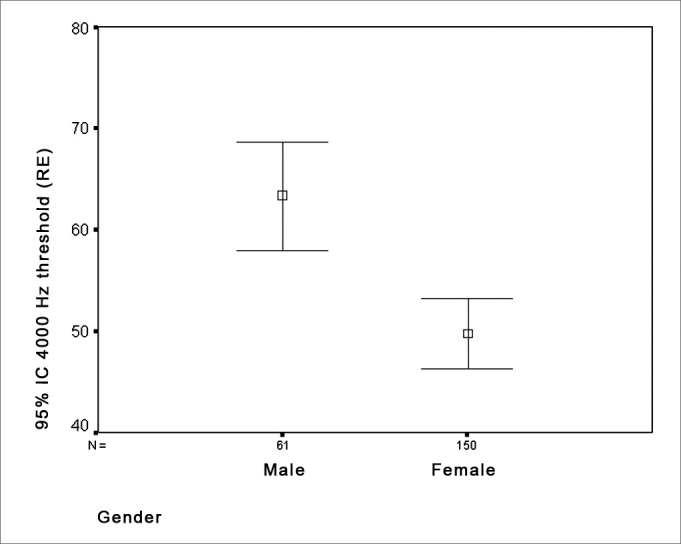
Distribution of the gender relation - RE threshold - RE = right ear

The investigation of threshold differences according to gender showed that there was a statistically significant threshold difference at 4KHz, where men had lower thresholds in both ears compared to women (Figuras 3 e 4).

**Figure 4:**
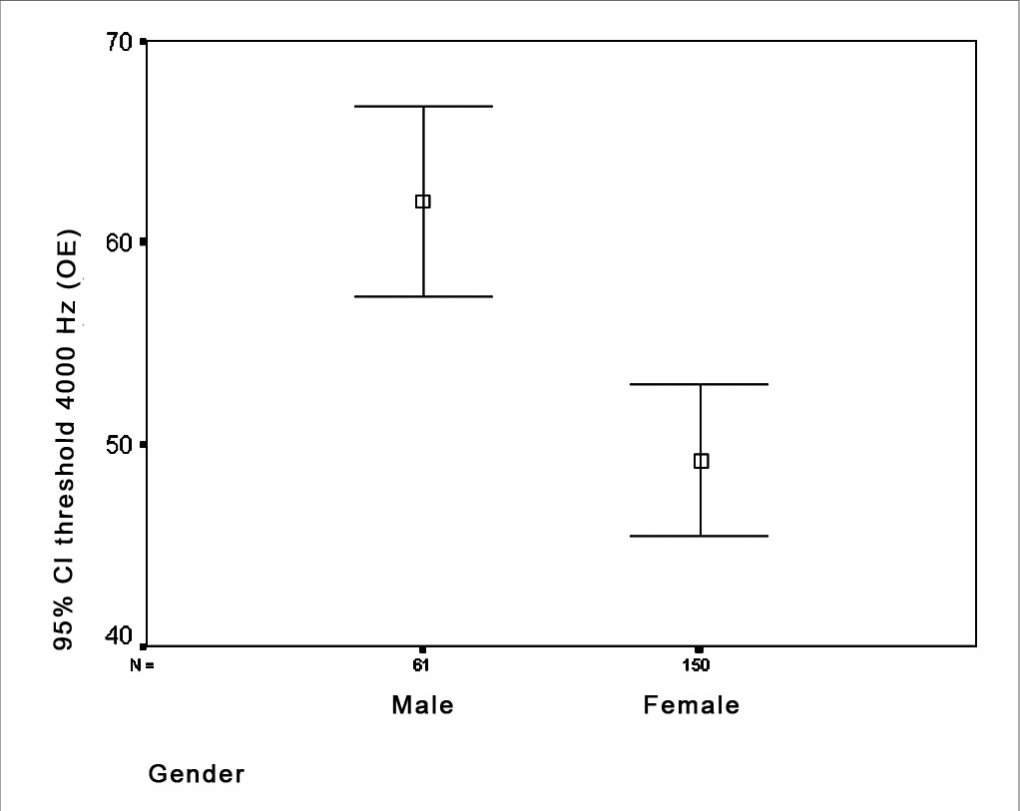
Distribution of the gender relation - LE threshold - LE = left ear

Various studies reveal lowered tone thresholds at high frequencies in men. Moscicki et al.^24^ studied 935 men and 1,358 women aged over 60 years, and reported loss of auditory sensitivity mostly at high frequencies (2 to 8 kHz), where thresholds in men were lower than in women. Russo^18^ also found greater increases in tone thresholds at high frequencies (4 to 8 kHz) in elderly men compared to elderly women. Mazeola et al.^17^ studied 30 elderly patients aged 67 to 93 years and found a statistically significant difference between sexes at 3 and 4 kHz, where men had a worse performance than women at these frequencies.

**Figure 5:**
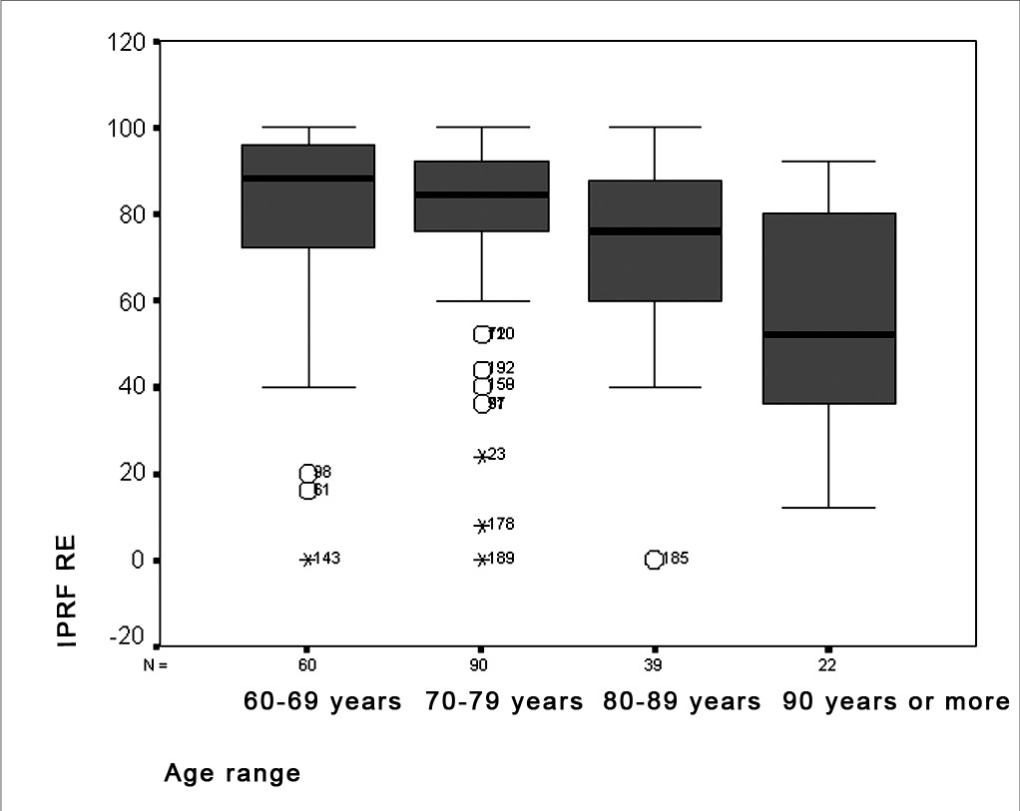
Distribution of the percent index of speech recognition in the RE according to the age group - RE = right ear

**Figure 6:**
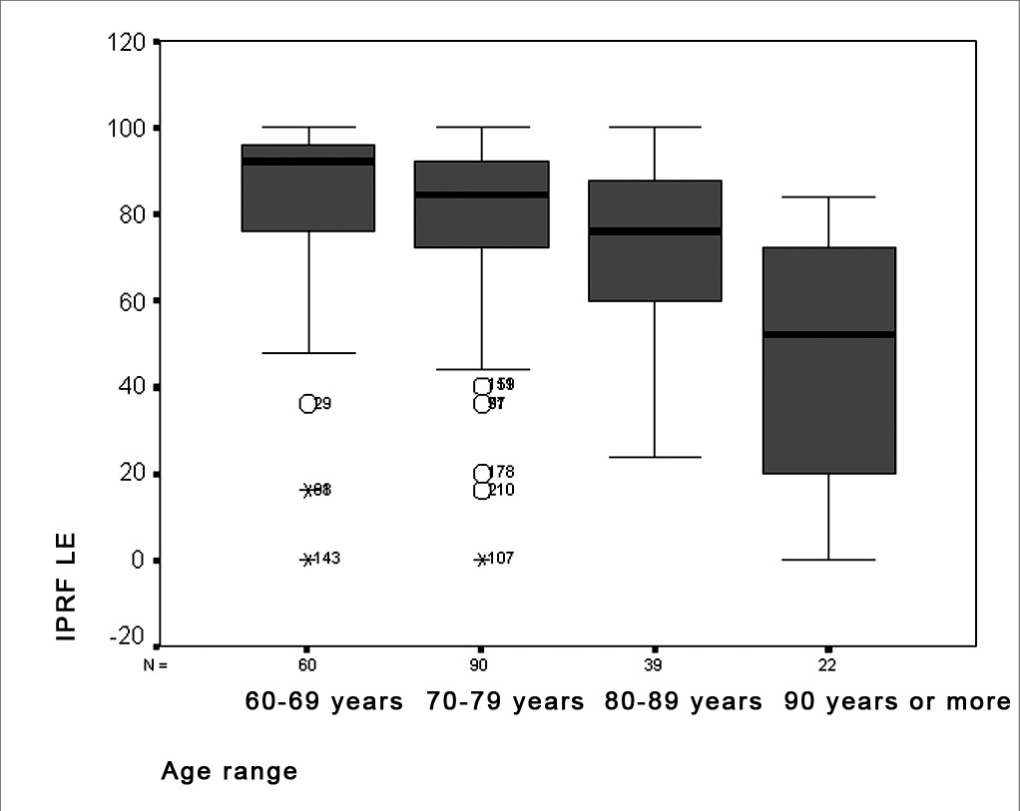
Distribution of the percent index of speech recognition in the LE according to the age group - LE = left ear

Results of the percent index of speech recognition revealed a significant performance difference in age groups where discrimination values declined with increasing age (Figures 5 and 6). This may be a result of auditory aging, generally characterized by threshold sensitivity loss and a lower ability to understand speech at a comfortable intensity. Shinohara et al. report that individuals with sensorineural hearing loss have an abnormal perception of loudness, which may interfere in speech discrimination.^25^ Furthermore, data in literature suggests that elderly persons have serious difficulties in understanding speech, as observed in speech audiometry.^18^ The average percent index of speech recognition value was 75.73% for the right ear and 75.50% for the left ear. A variation of 12% to 100% between both ears was observed. These values are lower than those reported in literature, in which the average value is 87%, varying from 34% to 100%. This finding may be due to poor discrimination associated with sloping pure tone curves and increased degrees of hearing loss, as seen in this study.^19^

## CONCLUSION

We may conclude that the degree of hearing loss progresses gradually with age, where audiometry shows sloping hearing loss with greater impairment at high frequencies in the 80 to 89 years age group, leveling in individuals aged over 90 years.

Men had lower thresholds at 4000Hz compared with women.

There was a gradual decrease in speech understanding responses with increasing age.
